# Cav3-KCa3.1 complex enhances detection of facilitating parallel fiber inputs in cerebellar Purkinje cells

**DOI:** 10.1186/1471-2202-13-S1-P142

**Published:** 2012-07-16

**Authors:** Jordan DT Engbers, Ray W Turner

**Affiliations:** 1 Hotchkiss Brain Institute, University of Calgary, Calgary, Alberta, Canada

## 

The ability for a neuron to distinguish important input signals from background noise is essential for proper circuit function. Several mechanisms exist to enhance signal detection. For example, short-term facilitation enhances burst transmission, while information from single spikes is transmitted more effectively through synapses that exhibit depression [1]. However, in the case of cerebellar Purkinje cells, where thousands of parallel fiber (PF) synaptic inputs impinge on the dendritic tree, postsynaptic mechanisms must play a role in increasing signal-to-noise ratio. Specifically, mechanisms must exist in Purkinje cells that allow transmission of granule cell bursts carrying sensory information while filtering out background input. Recently, we demonstrated that low voltage activated Cav3 calcium channels form a complex with intermediate conductance calcium-activated potassium channels (KCa3.1) in Purkinje cells [2]. We proposed that the Cav3-KCa3.1 complex enhances signal detection capabilities by selectively suppressing non-facilitating background inputs while allowing facilitating parallel fiber inputs to generate Purkinje cell spike output.

We used a combination of modeling and experimental methods to examine the role of the Cav3-KCa3.1 complex in simultaneously enhancing detection of parallel fiber bursts and suppressing background inputs. The Cav3-KCa3.1 complex was simulated using a calcium-diffusion model with 10 hemispherical compartments around the Cav3 channel and the KCa3.1 channel placed within 50 nm of the calcium source. The Cav3-KCa3.1 complex was included in a single-compartment membrane model with HCN and synaptic conductances to determine its effect on excitatory postsynaptic potential (EPSP) summation. Whole-cell current-clamp recordings of Purkinje cells were also made from acute slices of rat cerebellum (P18-P25). Parallel fibers were stimulated using a monopolar stimulating electrode in the cerebellar molecular layer.

We simulated 5 pulse 50 Hz trains of facilitating and non-facilitating EPSPs in the model. The Cav3-KCa3.1 complex reduced the summation of both sets of inputs. However, the facilitating inputs achieved a higher degree of summation than non-facilitating inputs, even though charge transfer for both trains was identical. Examination of the KCa3.1 current showed that synaptic facilitation compensated for the increase in KCa3.1 activation during the input train, allowing EPSPs to summate to higher voltages. When the Cav3-KCa3.1 complex was not included in the model, facilitating and non-facilitating inputs reached similar degrees of summation. Our computational results were confirmed by our current clamp recordings. Facilitating inputs evoked by direct PF stimulation generated more depolarization and spikes in Purkinje cells than non-facilitating simulated EPSPs (simEPSPs). During trains of simEPSPs, a supralinear increase in the rate of EPSP decay was observed for consecutive inputs, indicating an increased recruitment of hyperpolarizing current during the train. Application of Ni^2+^ (100 μM) to block Cav3 current removed this supralinear increase in rate of decay, confirming that the Cav3-KCa3.1 complex dynamically changes temporal summation during repetitive input trains.

Our results demonstrate that the Cav3-KCa3.1 complex significantly affects the temporal summation of synaptic inputs. Specifically, the Cav3-KCa3.1 complex allows facilitating inputs from single PF sources to summate and generate spike output in Purkinje cells, while background inputs are quickly suppressed, improving the ability to respond to important sensory information.
